# Supplementary material for: From sequence to enzyme mechanism using multi-label machine learning
Source: BMC Bioinformatics. 2014 May 19;15:150. doi: 10.1186/1471-2105-15-150 (PMC4229970; doi:10.1186/1471-2105-15-150)
Supplement: Additional file 2 — Java code of ml2db. Additional file ml2db_code.tar.gz contains the Java source code to run the multi-label machine learning experiments and save the results to database. The code’s Javadoc is included. [file 1471-2105-15-150-S2.zip › additional file 2/ml2db/ml2db/doc/test/graphviz/GraphVizArffTest.html]

GraphVizArffTest


---


|  |  |  |
| --- | --- | --- |
| **Overview** \| **Package** \| **Class** \| **Use** \| **Tree** \| **Deprecated** \| **Index** \| **Help** | |  |
| **PREV CLASS**   **NEXT CLASS** | **FRAMES**    **NO FRAMES**     **All Classes** |  |
| SUMMARY: NESTED \| FIELD \| CONSTR \| METHOD | DETAIL: FIELD \| CONSTR \| METHOD |  |


---


## test.graphviz Class GraphVizArffTest

```
java.lang.Object
  junit.framework.Assert
      junit.framework.TestCase
          test.graphviz.GraphVizArffTest
```

**All Implemented Interfaces:**: junit.framework.Test

---

``` public class GraphVizArffTest extends junit.framework.TestCase ```

Class

**Version:**
:   14 Apr 2012

**Author:**
:   Luna De Ferrari luna.deferrari@ed.ac.uk or
    luna.deferrari@st-andrews.ac.uk

---

**Constructor Summary**

`GraphVizArffTest()`


| **Method Summary** | |
| --- | --- |
| `static GraphVizArff` | `getGraphVizArffTest()` |
| `static GraphVizArff` | `getGraphVizArffTrain()` |
| `void` | `testGetDot1()` |
| `void` | `testGetDot2()` |

**Methods inherited from class junit.framework.TestCase**

`countTestCases, getName, run, run, runBare, setName, toString`

**Methods inherited from class junit.framework.Assert**

`assertEquals, assertEquals, assertEquals, assertEquals, assertEquals, assertEquals, assertEquals, assertEquals, assertEquals, assertEquals, assertEquals, assertEquals, assertEquals, assertEquals, assertEquals, assertEquals, assertEquals, assertEquals, assertEquals, assertEquals, assertFalse, assertFalse, assertNotNull, assertNotNull, assertNotSame, assertNotSame, assertNull, assertNull, assertSame, assertSame, assertTrue, assertTrue, fail, fail, failNotEquals, failNotSame, failSame, format`

**Methods inherited from class java.lang.Object**

`equals, getClass, hashCode, notify, notifyAll, wait, wait, wait`

**Constructor Detail**

### GraphVizArffTest

```
public GraphVizArffTest()
```


**Method Detail**

### testGetDot1

```
public void testGetDot1()
```

---


### testGetDot2

```
public void testGetDot2()
```

---


### getGraphVizArffTest

```
public static GraphVizArff getGraphVizArffTest()
```

---


### getGraphVizArffTrain

```
public static GraphVizArff getGraphVizArffTrain()
```


---


|  |  |  |
| --- | --- | --- |
| **Overview** \| **Package** \| **Class** \| **Use** \| **Tree** \| **Deprecated** \| **Index** \| **Help** | |  |
| **PREV CLASS**   **NEXT CLASS** | **FRAMES**    **NO FRAMES**     **All Classes** |  |
| SUMMARY: NESTED \| FIELD \| CONSTR \| METHOD | DETAIL: FIELD \| CONSTR \| METHOD |  |


---
